# Optic radiation structure and anatomy in the normally developing brain determined using diffusion MRI and tractography

**DOI:** 10.1007/s00429-013-0655-y

**Published:** 2013-10-30

**Authors:** Michael Dayan, Monica Munoz, Sebastian Jentschke, Martin J. Chadwick, Janine M. Cooper, Kate Riney, Faraneh Vargha-Khadem, Chris A. Clark

**Affiliations:** 1 Imaging and Biophysics Unit, UCL Institute of Child Health, London, UK; 2 Wellcome Trust Centre for Neuroimaging, UCL Institute of Neurology, London, UK; 3 School of Medicine, University of Castilla-La Mancha, Albacete, Spain; 4 Developmental Cognitive Neuroscience Unit, UCL Institute of Child Health, London, UK; 5 Free University, Cluster of Excellence “Languages of Emotion”, Berlin, Germany; 6 Research Department of Cognitive, Perceptual, and Brain Sciences, UCL Institute of Behavioural Neuroscience, London, UK; 7 Murdoch Childrens Research Institute, Melbourne, Australia; 8 Neurosciences Unit, Mater Children’s Hospital, South Brisbane, Australia

**Keywords:** Probabilistic tractography, Diffusion imaging, DTI, Optic radiation, Maturation, Brain, Meyer’s loop, Lateral geniculate nucleus, Epilepsy surgery, Neurosurgery planning

## Abstract

The optic radiation (OR) is a component of the visual system known to be myelin mature very early in life. Diffusion tensor imaging (DTI) and its unique ability to reconstruct the OR in vivo were used to study structural maturation through analysis of DTI metrics in a cohort of 90 children aged 5–18 years. As the OR is at risk of damage during epilepsy surgery, we measured its position relative to characteristic anatomical landmarks. Anatomical distances, DTI metrics and volume of the OR were investigated for age, gender and hemisphere effects. We observed changes in DTI metrics with age comparable to known trajectories in other white matter tracts. Left lateralization of DTI metrics was observed that showed a gender effect in lateralization. Sexual dimorphism of DTI metrics in the right hemisphere was also found. With respect to OR dimensions, volume was shown to be right lateralised and sexual dimorphism demonstrated for the extent of the left OR. The anatomical results presented for the OR have potentially important applications for neurosurgical planning.

## Introduction

Among the primordial senses lies vision, provided by the central nervous system through the central visual pathway. The latter extends from the eye to the occipital lobe and the optic radiation (OR) is at its posterior part, a white matter bundle emerging from the lateral geniculate nucleus (LGN) and ending in the primary visual cortex. After leaving the LGN anteriorly passing over the roof of the lateral ventricle temporal horn and turning inferolaterally towards the pole of the temporal lobe, the inferior fibers of the OR make a loop to return posteromedially (Ebeling and Reulen 1988; Wahler-Luck et al. 1991; Yasargil et al. 2004). This characteristic sharp curve around the tip of the lateral ventricle temporal horn is called Meyer’s loop (ML), named after one of the first anatomists who described it (Meyer 1907). A remarkable feature of the visual pathway is that it conserves retinotopic organisation such that the visual field on one side is mostly transmitted within the OR of the contralateral hemisphere. It has been shown through dissection studies that the OR is one of the first white matter pathways to mature, reaching adult myelin concentration before the age of 3 years (Kinney et al. 1988).

Diffusion tensor imaging (DTI) is an imaging modality that allows, in vivo, the study of the microstructure of white matter tracts. The technique is based on the observation that brain microstructure impedes the diffusion of water molecules (Le Bihan 2003). The extent to which these molecules travel via diffusion (their diffusivity) is evaluated in a discrete number of directions with the aim of characterising the diffusion profile in three dimensions. Orientation preference in this profile originates from the directional nature of brain microstructure, arising in particular in the white matter. Specific white matter tracts can be isolated or ‘virtually dissected’ using tractography that identifies connections between defined brain regions (see for example Ciccarelli et al. 2008; Lazar 2010). This method is based on the fundamental assumption that the direction(s) of the underlying fascicle(s) coincides with the peak(s) in the measured diffusion profile. DTI is well suited to study the maturation of specific white matter tracts which cannot otherwise be easily distinguishable on conventional MRI. DTI metrics provide quantification of different aspects of diffusion which relate to the underlying microstructure; these include the fractional anisotropy (FA), mean diffusivity (MD), axial diffusivity (λ_∥_) and radial diffusivity (λ_⊥_) as detailed in “Maturation, lateralization and sexual dimorphism as shown by DTI metrics”. For this reason, numerous studies have used this imaging modality to investigate normal brain development (Schmithorst et al. 2002; Barnea-Goraly et al. 2005; Eluvathingal et al. 2007).

A number of studies have used tractography to reconstruct the OR in adults (Yamamoto et al. 2005; Wakana et al. 2007). Other studies have been performed to investigate OR maturation in neonates and infants, also based on tractography (Dubois et al. 2008a; Berman et al. 2009) or by manual ROI delineation (Gao et al. 2009). They showed an increase of FA and a decrease of λ_∥_, λ_⊥_ and MD with age. Studies on brain maturation in childhood and adolescence indicate ongoing, although less rapid changes in DTI metrics (Lebel and Beaulieu 2011). However, only one study was found showing the support for OR microstructural changes in children and adolescents (increase of FA and decrease of MD), in a small cohort (5 males and 8 females) (Govindan et al. 2008).

**Fig. 1:**
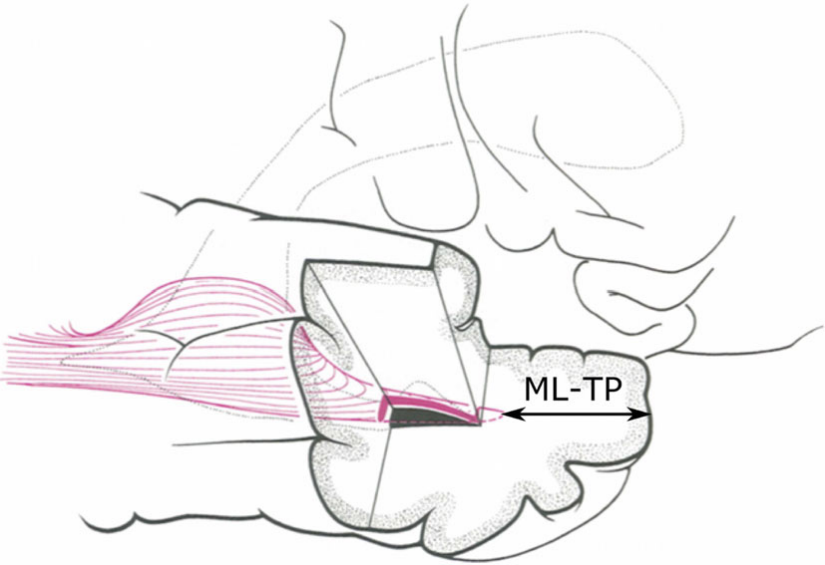
Temporal lobectomy, reproduced with permission from Ebeling and Reulen (1988). Temporal lobectomy is a procedure during which ML is at risk of damage. Tractography can estimate the distance separating ML to the TP, ML–TP. *ML* Meyer’s loop, *TP* Temporal pole

A further application of OR tractography is for neurosurgical planning of temporal lobectomy for intractable epilepsy (Yogarajah et al. 2009). In this case, tractography can provide information concerning the distance from the temporal pole to the tip of ML (ML–TP), with the latter being at risk of damage during the procedure leading to visual field defect, as shown in Fig. 1 (Yasargil et al. 2004). Children and adolescents represent an important group as epilepsy surgery is often performed in this age range, but no data on anatomical distances in this group have been documented thus far.

The aim of this work was therefore to examine the maturation of the OR in childhood and adolescence in a much larger sample (*N* = 90, 5–18 years) than hitherto examined to elucidate and characterise more fully structural trajectories of the OR and associated anatomical distances, including investigation of possible age dependencies, hemisphericity and gender effects.

## Methods

### Subjects

**Fig. 2:**
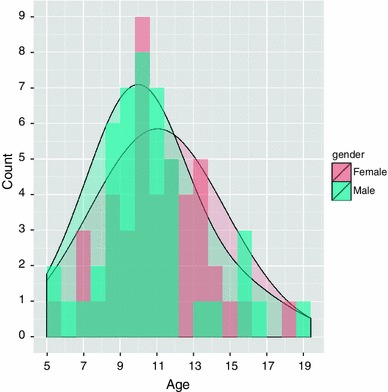
Age histogram and distribution relative to the subjects. The histogram origin was set at age of 5 years and had bin size of 0.8 years with females indicated in *light red* and males in *light blue*. Two separate normal distributions were fitted to the age data of females and males, and represented with the same choice of colours

The study of the OR was carried out on data acquired at Great Ormond Street Hospital, London, UK. This work was granted ethical approval by the local ethics committee. Ninety healthy children and adolescents without any known medical condition took part in the study. Informed consent was obtained in all subjects before their participation. The cohort included 46 males and 44 females with a mean age of 10.8 ± 2.6 years (Fig. 2). The age range extended from 5^1/2^ to 18^1/2^ years.

### Imaging

Each participant underwent a DTI protocol on a Siemens Avanto 1.5T clinical system (Siemens Healthcare, Erlangen, Germany), using a self-shielding gradient set with maximum gradient strength of 40 mT·m^−1^ and standard ‘birdcage’ quadrature head coil. Echoplanar diffusion-weighted images were acquired for an isotropic set of 20 noncollinear directions, using a weighting factor of 1,000 s·mm^−2^, along with a T2-weighted (*b* = 0) volume, and with a repetition factor (or NEX, Number of EXcitations) of 2 to increase SNR. This protocol was repeated three times in a single scan session, and the data merged together without averaging. 45 contiguous axial slices of thickness 2.5 mm were imaged, using a field of view of 240 × 240 mm^2^ and 96 × 96 voxel acquisition matrix, for a final image resolution of 2.5 × 2.5 × 2.5 mm^3^. Echo time was 89 ms and repetition time was 6,300 ms. In addition, a T1-weighted 3D FLASH structural image was acquired using 176 contiguous sagittal slices, a 256 × 224 mm^2^ field of view and 1 × 1 × 1 mm^3^ image resolution. Echo time in this case was 4.9 ms, and repetition time was 11 ms. Overall scan time for the acquisition of these sequences was approximately 19 min.

### Data processing

### Tractography

The tractography method chosen was the Probability Index of Connectivity (PICo) algorithm (Parker et al. 2003), as implemented in Camino, which was used in a previous study to reconstruct the OR (Yogarajah et al. 2009). Briefly, the PICo method associates the main diffusion direction *e*_1_ measured in each voxel to a probability density function (PDF) accounting for the uncertainty in *e*_1_. Deterministic tracking from each voxel of the seed ROI was repeated many times via a Monte Carlo simulation. Before a given tracking iteration, the PDF associated to the local main diffusion direction *e*_1_ in each voxel was sampled, resulting in a unique distribution of diffusion directions within the whole brain. One tracking iteration produced a track according to this distribution. The result after a total of *N* = 10,000 iterations was a probability map in which the PICo value in each voxel was equal to the number of iterations resulting in a track going through that voxel divided by *N*. A probability map was obtained for each seed voxel and the final map was computed as the maximum intensity projection of these probability maps (i.e. its value at one location was the maximum of the probability map values at that location).

**Fig. 3:**
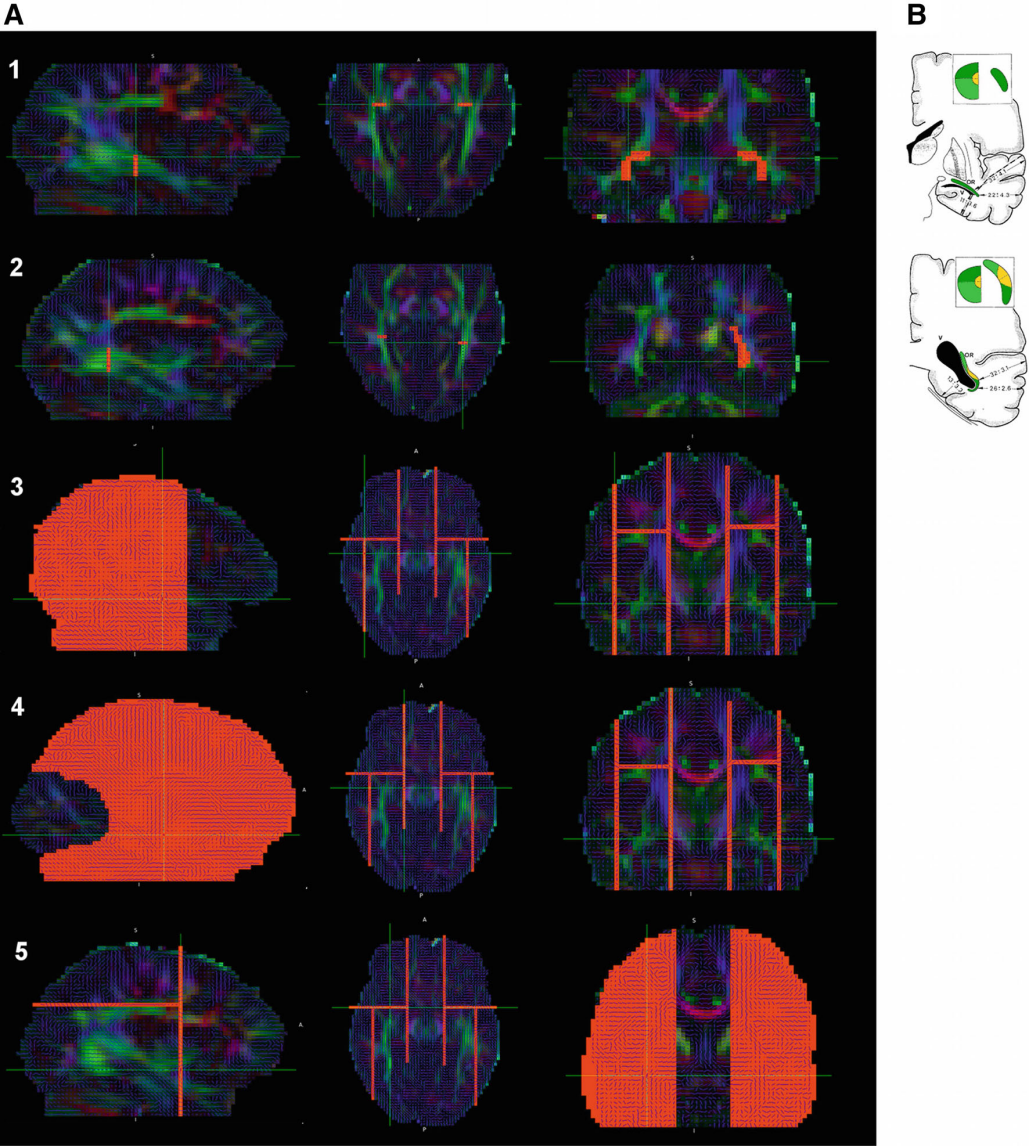
Sagittal, axial and coronal slices of a subject FA map coloured according to the principal diffusion direction (*green* anterior–posterior, *blue* superior–inferior, *red* left–right) illustrating the placement of the ROIs used in the tractography analysis (shown in *orange*) **a** and the documented positions of the OR in the coronal plane as indicated by and reproduced with permission from Ebeling and Reulen (1988) **b**. The seed ROI was placed in a single coronal slice anterolaterally to the LGN and included solely voxels coloured in green to intersect fibers running in the anterior–posterior directions (*1*). The waypoint ROI was drawn laterally and inferiorly to the lateral ventricles at the level of the trigone (*2*). Three main exclusion ROIs were systematically used: one placed laterally (*3*), another medially (*4*) and finally one anteriorly (*5*) to the OR

**Fig. 4:**
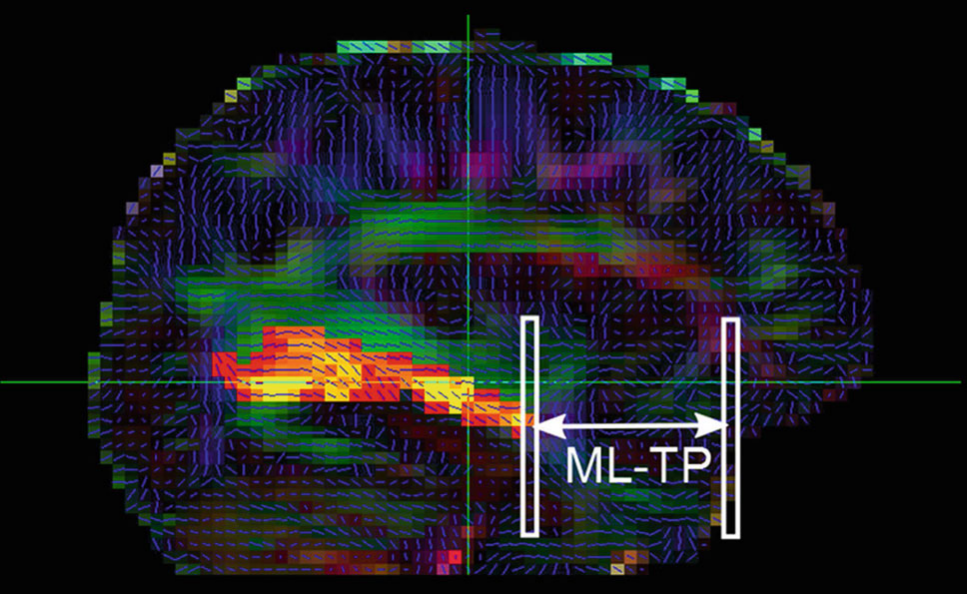
ML–TP distance, as measured from tractography results. The image showed is one sagittal slice of a subject FA map with the same colour coding as in Fig. 3. Overlaid using a hot colour scale is the OR reconstruction. The ML–TP distance was calculated as the length separating the most anterior voxel from the OR reconstruction and the most anterior voxel of the temporal lobe

**Fig. 5:**
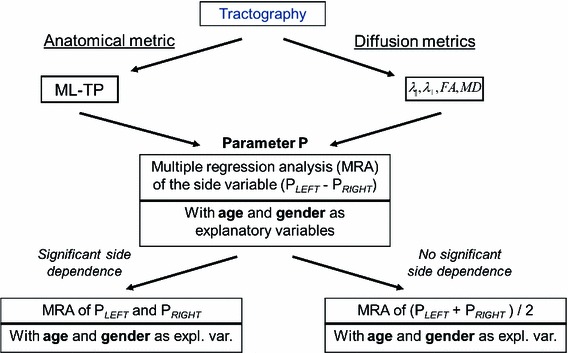
Summary of the statistical analysis on the anatomical and diffusion metrics. To avoid the statistically complex repeated measurement problem, the side difference variable *P*_HEMI_ = *P*_LEFT_ − *P*_RIGHT_ between the value of the parameter *P* in the left and right hemisphere was first submitted to an MRA with variables age and gender and their interaction term. If one of these variables showed a significant effect, a second MRA was conducted separately for *P*_LEFT_ and *P*_RIGHT_ in each hemisphere; otherwise a *t* test was performed on the side variable *P*_HEMI_. If the *t* test demonstrated that *P*_HEMI_ was significantly different from zero, then as previously a second MRA was conducted separately for *P*_LEFT_ and *P*_RIGHT_. Otherwise if *P*_HEMI_ was not significantly different from zero then a second MRA was performed on the average of *P* between the two hemisphere, (*P*_LEFT_ + *P*_RIGHT_)/2

In order to perform tractography for the OR, ROIs were defined in the colour-coded FA maps on which was overlaid the main diffusion direction. The seed region—from which all the tracts are initiated—was placed slightly anterolaterally to the LGN to include voxels of ML where the vector directions were consistent with the expected track orientation as described previously in the studies of Nilsson et al. (2007), Govindan et al. (2008), Yogarajah et al. (2009). Furthermore, this ROI was selected in a region of high FA corresponding to a bright green area in the colour-coded map. It consisted of 16 voxels, all selected within the same coronal slice as shown in Fig. 3-1. A waypoint ROI—selecting tracts only going through this region—was also drawn on a frontal slice in a similar green, high-intensity region to include the sagittal stratum of which the OR is a part (Fig. 3-2). Several exclusion ROIs—eliminating tracts intersecting them—were defined solely according to the main diffusion directions to exclude neighbouring tracts to ML. These ROIs were placed laterally (Fig. 3-3) to prevent tracking of the acoustic radiation, medially (Fig. 3-4) to avoid the generated tracts following the anterior commissure and forceps major and anteriorly (Fig. 3-5) to prevent tracking of bundles continuing along the inferior occipito-frontal fasciculus and uncinate fasciculus. Finally, obvious extra artifactual tracts were removed by adding planar exclusion ROIs that intersected them.

Once the ROIs were created, the PICo algorithm was run without any angular threshold to account for the high curvature of ML and with a relatively low FA threshold, 0.1, to allow the tracts to reach the extremities of the OR ending in grey matter regions while avoiding a too high number of spurious tracts associated with voxels exhibiting high uncertainty in the diffusion directions. The maximum intensity projection of the probabilistic maps generated for each seed voxel was displayed with a linear scale, from red to yellow, and with a threshold of 1% to visually eliminate artifactual tracts without removing plausible OR bundle reconstructions.

### Maturation, lateralization and sexual dimorphism as shown by DTI metrics

Most of the parameters selected to quantify the microstructural characteristics of the segmented OR were derived from the DT. They were:

- the average FA
- the average MD
- the track volume (in terms of number of voxels)
- the average axial diffusivity λ_∥_
- the average radial diffusivity λ_⊥_.

The computation of their values was achieved using a custom MATLAB^®^ script. It consisted in selecting only voxels whose probability was greater than the chosen PICo threshold of 1 %, which resulted in the creation of a binary mask. This threshold was chosen as it was considered to correspond to the most visually convincing reconstruction of the OR. The number of non-zero voxels in such masks gave the estimated OR volume for this probability threshold. The next step was to select the area corresponding to the OR in each of the DTI metrics volume (e.g. the FA volume) and keep only voxels with PICo value >1 %. This step was carried out by a voxel-by-voxel multiplication with the binary mask previously computed. An average over the resulting thresholded area was then calculated and provided the DTI metric mean corresponding to a 1 % PICo threshold.

### Neuroanatomical distances of the OR

To assess the spatial location of ML and compare the results with reference dissection studies, the distance from its tip to the temporal pole (ML–TP) was measured. The tip of ML was identified by visual inspection of the PICo maps displayed with a threshold of 1 %. The position of the most anterior voxel belonging to the OR was chosen as the location of the tip of ML. The location of the TP was evaluated from the colour-coded FA maps and chosen as the coronal slice coinciding with the vertical portion of the contour including the TP and the beginning of the frontal lobe. In cases where several coronal plane positions along the anterior–posterior axis fit this criterion, the most anterior position was systematically selected. The ML–TP distance was computed as the difference between the tip of ML and the TP, as illustrated in Fig. 4.

### Statistical analysis

The results obtained from both the maturation and anatomical mapping studies were subjected to a multiple regression analysis (MRA) in SPSS^®^ with respect to cerebral hemisphere (right/left), age and gender and are illustrated in Fig. 5. The statistical significance was generally evaluated with the associated *p* value considered to be significant when less than 5 %.

The side effect (i.e. lateralization) was first tested. To circumvent the statistically complex repeated measurement problem, the dependence evaluation was performed on a left/right difference variable (*results for right* OR − *results for left* OR). An MRA was applied with variables age and gender, together with the associated interaction term. The model was first evaluated for significant influence of the interaction term and if demonstrated to be negligible, re-run with only the age and gender variables. If the model demonstrated an age*gender, age or gender effect on the left/right difference variable, then the data were analysed separately in each hemisphere as it would be statistically inaccurate to combine them. If no such effect was shown to be significant, a one-sample *t* test was performed on the same difference variable. If the difference variable proved to be significantly different from zero, then the data were also separated according to hemisphere, otherwise they were averaged over each hemisphere and the results were given as mean ± standard error (SE).

Finally, the processed data (averaged across hemispheres or calculated separately in each hemisphere) were tested for gender and/or age effect again using an MRA. If the interaction term was found to be negligible, the gender effect was then evaluated. If this was shown to be significant, the data were analysed for each gender, otherwise it was averaged across gender. If demonstrated to be significant, the age effect was investigated according to a linear regression model.

## Results

### Tractography reconstruction

**Fig. 6:**
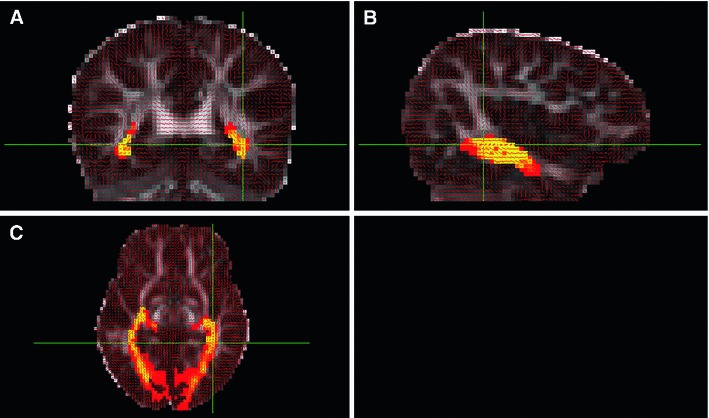
Example of probabilistic map of the OR to which is applied a PICo threshold of 1 % represented in a coronal (**a**), sagittal (**b**) and axial (**c**) plane. The OR reconstruction is displayed in a linear hot colour scale ranging from just above 0 (*red*) to 1 (*yellow*). The background FA map is shown in greyscale and the main diffusion directions in each voxel are shown as small red segments

An example of a probability map with PICo threshold 1 % obtained for one representative subject is shown in Fig. 6. The greyscale background image is an FA map, while the red segments indicate the main diffusion direction. The value of the tractography probability in each voxel is displayed on a linear scale from just above 0 in pure red (one track went through that voxel) to 1 in pure yellow (all the tracts generated passed through that voxel). The reconstruction of the OR was satisfactory in all subjects. The distinctive shape of ML was clearly visible in all subjects, and the entirety of the OR—from the LGN to the occipital lobe—could be systematically segmented. The reconstructed tracts run along the lateral ventricles as expected, before curving medially towards the mid-sagittal plane. Also the exclusion masks were particularly efficient at removing most artefactual tracts as only very few of these were visible in the final segmentation.

### Maturation, lateralization and sexual dimorphism as shown by DTI metrics

#### Fractional anisotropy (FA)

**Fig. 7:**
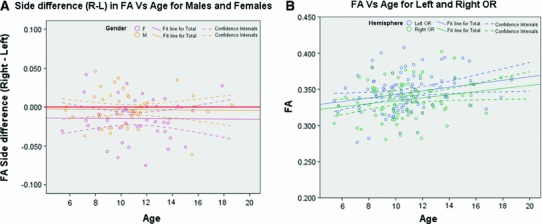
Changes with age of FA side difference variable (FA *Right*–FA *left*) (**a**) and FA (**b**). **a** The FA side difference variable is shown in *purple* for females and in *orange* for males. The value corresponding to no difference between left and right hemisphere is represented by a *bold red horizontal line*. **b** FA values of the left OR are shown in *blue* and for the right OR in *green*. **a**, **b** Linear regressions are displayed as *solid lines*, while their associated confidence intervals are demarcated with *dashed lines*

The difference variable of the mean FA between hemisphere (*right*–*left*) did not demonstrate an age effect but showed a gender effect (*p* < 0.05) (see Fig. 7a); therefore, FA values were analysed in each hemisphere separately. The MRA applied on the mean FA of each side demonstrated a significant age effect (*p* < 0.05 in each hemisphere) as illustrated in Fig. 7b. The estimated slopes from the linear regression between FA and age were 0.0024 with 95 % confidence range [0.0004, 0.0045] and 0.0021 with 95 % confidence range [0.0001, 0.0041] for the left and right OR, respectively.

#### Mean diffusivity (MD)

The MD lateralization variable *right*–*left* did not demonstrate any gender or age effect. A *t* test showed it was significantly less than 0 (*p* < 0.0005) and the statistical analysis was thus conducted separately on each side. In the left hemisphere, no age or gender effect was found (*p* = 0.27 and *p* = 0.09, respectively), while in the right hemisphere both age and gender effects were shown, with both *p* values <0.025. As a result, the analysis was separated by gender in each hemisphere.

**Fig. 8:**
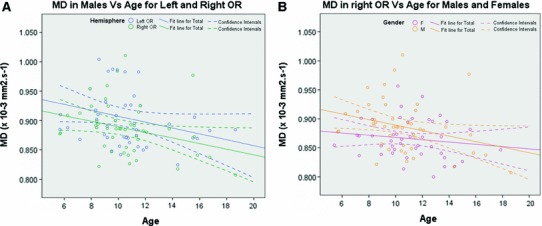
Changes with age of MD in the left and right OR of males (**a**) and in the right OR of both males and females (**b**). **a** MD values for males are shown in *blue* for the left OR and in *green* for the right OR. **b** MD values in the right OR are shown in *orange* for males and in *purple* for females. **a**, **b** Linear regressions are displayed as *solid lines*, while their associated confidence intervals are demarcated with *dashed lines*

In both hemispheres, no significant age effect was found for females (*p* = 0.69 and *p* = 0.34 for left and right side, respectively). It was shown to be significant for males in the right hemisphere (*p* < 0.05) as shown in Fig. 8a, but did not reach significance in the left hemisphere (*p* = 0.075). The linear regression carried out subsequently on MD as a function of age for males gave mean slopes and 95 % confidence intervals of −0.0050 [−0.0105, 0.0005] × 10^−3^ mm^2^·s^−1^ in the left hemisphere and −0.0048 [−0.0094, −0.0001] × 10^−3^ mm^2^·s^−1^ in the right. For comparison in the latter hemisphere, the mean slopes and 95% confidence intervals for females were −0.0020 [−0.0061, 0.0021] × 10^−3^ mm^2^·s^−1^, as shown in Fig. 8b.

#### Axial diffusivity

The MRA applied to the side difference in λ_∥_ showed no significant age or gender effect. A one-sample *t* test on the same variable demonstrated a significant hemisphere effect (*p* < 0.0000005), with λ_∥_ being greater in the left hemisphere. As a consequence, λ_∥_ values were separated into hemisphere categories for further analysis.

In the left hemisphere, a significant age*gender interaction effect was found for λ_∥_ (*p* < 0.05) while in the right hemisphere a significant gender effect was found (*p* < 0.025) (but no age effect, *p* = 0.25). The statistical analysis was thus separated according to both gender and hemisphere.

**Fig. 9:**
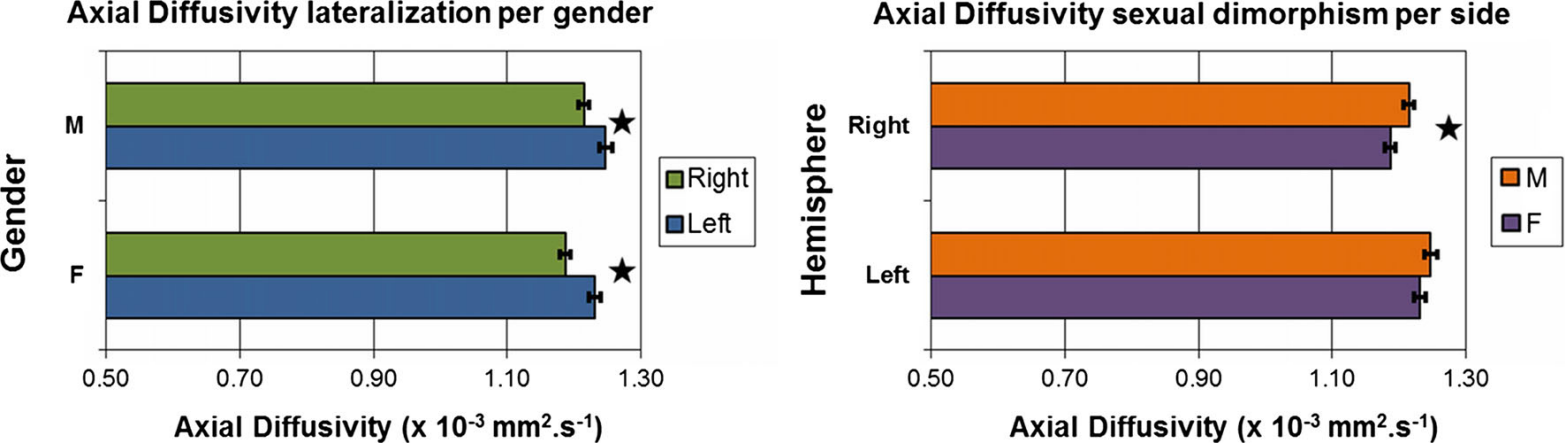
Axial diffusivity values showing side and gender differences. On the *left*, the values are separated by gender to compare results obtained in the *right* (*green*) and *left* (*blue*) hemispheres. On the *right*, the values are separated by hemisphere to compare results obtained in males (*orange*) and females (*purple*). In both graphs, statistically significant differences (at the *p* < 0.05 level) are indicated by *stars*

No age effect was demonstrated for λ_∥_ either in the left hemisphere (*p* = 0.08 for males and 0.22 for females) or in the right (*p* = 0.20 for males and 0.92 for females). λ_∥_ was thus averaged across age. The mean values ± SE for females and males were, respectively, 1.231 ± 0.009 and 1.247 ± 0.009   × 10^−3^ mm^2^·s^−1^ in the left hemisphere and 1.187 ± 0.008 and 1.215 ± 0.008   × 10^−3^ mm^2^·s^−1^ in the right hemisphere, as illustrated in Fig. 9.

#### Radial diffusivity

λ_⊥_ was also found to be dependent on hemisphere. No age or gender effect was found in the side difference variable and a *t* test demonstrated a significant side effect (*p* < 0.025), with λ_⊥_ being larger in the left hemisphere.

**Fig. 10:**
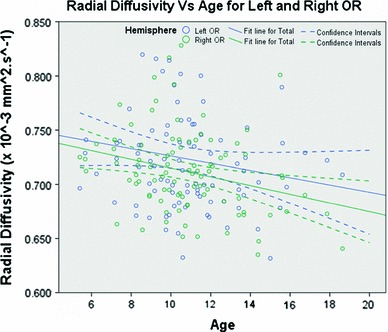
Radial diffusivity as a function of age in the left and right OR. Values for the left OR are shown in *blue* and values for the right OR in *green*. Linear regressions are displayed as *solid lines*, while their associated confidence intervals are demarcated with *dashed lines*

In the left OR, no gender or age effect was found (with *p* = 0.13 and 0.18, respectively). In the right OR, no gender effect was found (*p* = 0.24), but an age effect was demonstrated (*p* < 0.025) as shown in Fig. 10. The linear regression gave mean slopes and 95 % confidence intervals of −0.0027 [−0.0077, 0.0022]   × 10^−3^ mm^2^·s^−1^ in the left hemisphere and −0.0054 [−0.0096, 0.00013] × 10^−3^ mm^2^·s^−1^ in the right.

### Neuroanatomical mapping

#### Volume

The application of the MRA to the side difference (*right*–*left*) of the OR volume calculated with a PICo threshold of 1 % did not show any age or gender effect. A subsequent one-sample *t* test on this difference variable demonstrated that it was significantly greater than 0 (*p* < 0.00000005). Further analysis was then conducted separately on the two hemispheres.

**Fig. 11:**
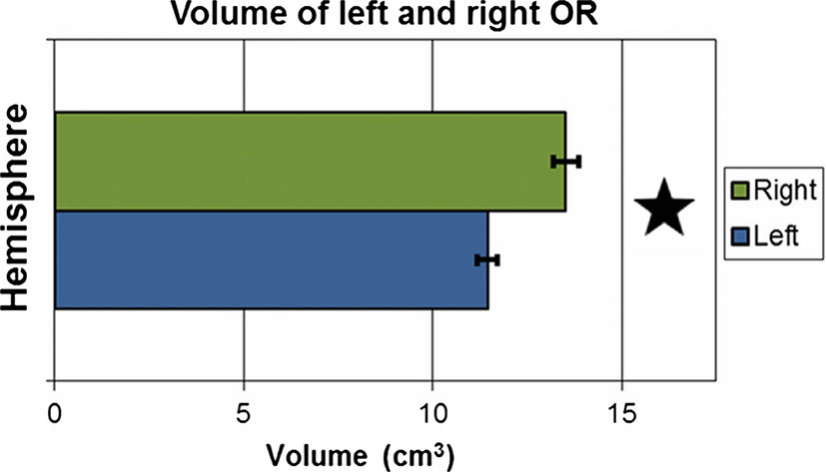
Volume in cm^3^ in the left OR and right OR. A *star* indicates a significant side effect (*p* < 0.05)

After being differentiated with respect to hemisphere (left and right), each of the side variables was tested for gender and age effect. No evidence for a gender effect from the MRA was found, nor was an age effect observed for either hemisphere. Therefore, the track volumes were averaged across age. The values of the volume computed in the left and right OR (mean ± SE) were 11.45 ± 0.27 and 13.53 ± 0.34 cm^3^, respectively. The significant side effect is illustrated in Fig. 11.

#### Anatomical measurements

The difference variable between hemispheres (*right* and *left*) for the ML–TP distance did not show any gender or age effect. The *t* test demonstrated that it was significantly different from zero (*p* < 0.05) with mean difference of 1.2 mm and 95% confidence range of [−2.3, −0.1] mm, i.e. the ML–TP distance showed a side effect with ML–TP being smaller on average in the right hemisphere. Accordingly, the ML–TP measurements were therefore analysed separately for each hemisphere.

**Fig. 12:**
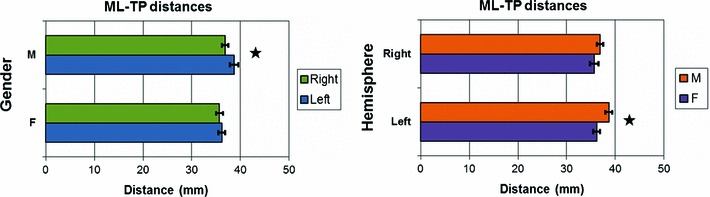
ML–TP distances showing side (*left*) and gender (*right*) differences. On the *left*, the values are separated by gender to compare results obtained in the *right* (*green*) and *left *(*blue*) hemisphere. On the *right*, the values are separated by hemisphere to compare results obtained in males (*orange*) and females (*purple*). In both graphs, statistically significant differences (at the *p* < 0.05 level) are indicated by *stars*

After being processed by the MRA model, described in “Statistical analysis”, the ML–TP distance in the left hemisphere did not demonstrate any age effect (*p* = 0.09), but did show a significant dependence on gender (*p* < 0.01). No age or gender effect was found in the right hemisphere. The anatomical measurements are summarised in Fig. 12 as well as in Table 1.

**Table 1:** ML–TP distances measured in the left and right hemispheres separated according to gender

Gender	Hemisphere	
	Left	Right
F	36.2 ± 0.7 (22.5, 45.0)	35.7 ± 0.9 (27.5, 45.0)
M	38.7 ± 0.7 (30.0, 47.5)	36.8 ± 0.7 (25.0, 50.0)

Measurements are indicated as Mean ± SE with the full range of values shown in brackets

## Discussion

### Maturation, lateralization and sexual dimorphism

#### Maturation

Before discussing the results obtained on the OR, a brief overview of the findings relative to other WM tracts is relevant to put into perspective our findings. An increase then decrease of grey matter volume (inverted U-shape) was reported in both cortical and subcortical areas (Gogtay et al. 2004; Luders et al. 2005; Sowell et al. 2003; Thompson et al. 2005), while white matter was shown to continuously increase in volume, roughly linearly (Paus et al. 2001; Bartzokis et al. 2010; Schmithorst and Yuan 2010; Paus 2010). Dissection studies have suggested that WM develops first within posterior sensory areas and at a later stage in anterior and temporal regions (Kinney et al. 1988; Flechsig Of Leipsic 1901; Yakovlev and Lecours 1967). It has been generally found that FA and MD increase and decrease, respectively, with age, especially due to a decrease in λ_⊥_ rather than changes in λ_∥_ (Schmithorst and Yuan 2010). Such observations from early childhood to the end of adolescence have been demonstrated in the internal capsule, corticospinal tract, inferior longitudinal fasciculus, splenium of the corpus callosum, superior longitudinal fasciculus, cingulum, inferior fronto-occipital fasciculus, uncinate fasciculus, external capsule, cingulum and left arcuate fasciculus (Schmithorst et al. 2002; Bonekamp et al. 2007; Eluvathingal et al. 2007; Schneider et al. 2004; Zhang et al. 2005; Ben Bashat et al. 2005; Lebel et al. 2008).

To the best of our knowledge, the only reference relative to OR maturation using DTI in children is Govindan et al. (2008) who showed an age effect for FA and MD in a small cohort of 13 children [5 males and 8 females aged 3–18 years (mean 9.1 ± 4.0 years)]. The latter study did not find any significant gender effect and did not investigate lateralization. With respect to adolescents and young adults, the only two studies known to address maturational changes, comparing characteristics of the OR between that group and older volunteers, were a study of FA and MD in 19 subjects aged 19–39 years and 12 subjects aged 40–65 years in Lee et al. (2009), and FA between 15 subjects aged 14–21 years and 33 subjects aged 22–64 years in Schneiderman et al. (2007). None of these studies found a significant age effect.

In our cohort of 90 subjects, we observed an increase in FA and a decrease in MD with age, in agreement with maturational changes found in other white matter tracts. We also observed evidence for lateralization and sexual dimorphism in DTI metrics. In addition, we demonstrated for the first time that the decrease in MD in the OR is mainly due to a decrease in λ_⊥_, similar to changes reported in other tracts, rather than a decrease in λ_∥_. The fact that we did not see an age effect in volume was not surprising. The OR and its direct afferent and efferent structures are known to mature very early in life. By the age of 9 months, the LGN has already reached adult morphology (De Courten and Garey 1982). The primary visual cortex reaches a volume of adult size by the age of 4 months, while synaptic density reaches adult levels after the age of 5 years (Huttenlocher et al. 1982). This is consistent with reports of curing loss of fixation in the squinting eye up to an age of 5–7 years (Assaf 1982; Awaya et al. 1973). Furthermore, the OR has been shown to be myelin mature before the age of 3 years (Kinney et al. 1988).

FA is thought to increase with the organisation and coherence of the underlying tracts, as well as with their density and myelin content (Beaulieu 2002). None of the DTI metrics are direct markers of myelin (Beaulieu and Allen 1994). However, λ_⊥_ has shown sensitivity to demyelination in mice (Song et al. 2002, 2005), and MD and FA have been correlated with myelin content according to an ex vivo imaging and histology study (Schmierer et al. 2007). λ_⊥_ has also been shown to be positively correlated to axonal caliber (Nair et al. 2005; Song et al. 2005), whilst λ_∥_ is thought to also increase with fiber coherence (Dubois et al. 2008b; Takahashi et al. 2000). Since MD is a linear combination of λ_⊥_ and λ_∥_ (MD = 1/3λ_∥_ + 2/3λ_⊥_), it is influenced by all these effects. The maturational processes discovered in our study are therefore not likely to be due to myelination but rather to local changes in the extracellular matrix, glial cell morphology (Beaulieu and Allen 1994; Trip et al. 2006) and axonal caliber, as suggested by the decrease in λ_⊥_, and also increase in fiber coherence and compactness (McGraw et al. 2002), with which the increase of FA is consistent.

#### Lateralization

Lateralization has been consistently reported in the cingulum (Gong et al. 2005; Trivedi et al. 2009; Bonekamp et al. 2007; Takao et al. 2011a, b; Verhoeven et al. 2010), superior longitudinal fasciculus (Buchel et al. 2004; Makris et al. 2005; Nucifora et al. 2005; Verhoeven et al. 2010; Takao et al. 2011a; Thiebaut de Schotten et al. 2011a) and arcuate fasciculus (Lebel and Beaulieu 2009; Barrick et al. 2007; Buchel et al. 2004; Catani et al. 2007; Parker et al. 2005; Takao et al. 2011a, b; Thiebaut de Schotten et al. 2011b; Verhoeven et al. 2010) while for other tracts the findings are inconsistent (Takao et al. 2011a). No significant age effect in lateralization is known to have been shown in specific WM tracts. A significant sex effect in lateralization has only been demonstrated in the left-lateralised arcuate fasciculus by Catani et al. (2007) (with significantly more streamlines in males).

To our knowledge, lateralization of DTI metrics in the OR has only been previously reported for FA. Left lateralization characterised by higher FA was shown by Xie et al. (2007) in children (10 males and 4 females aged 3.5–9 years) in which age effects were not investigated. The same leftward assymmetry in FA was demonstrated in a cluster of voxels within the OR by Park et al. (2004) in 32 adult males (aged 30–55 years, mean 44 ± 6.2 years) and by Kang et al. (2011) in 56 subjects (aged 21–37 years). Both left and right lateralization were found in different parts of the OR in a Tract-Based Spatial Statistics (TBSS) study conducted by Takao et al. (2011a) on 857 subjects (436 subjects with mean age 56.6 ± 9.8 years on one scanner and 421 subjects with mean age 55.6 ± 9.9 years on another), while Thiebaut de Schotten et al. (2011b) reported a rightward asymmetry in FA after tractography of the OR in 40 subjects (aged 18–22 years). In contrast with all these studies, we not only investigated lateralization but also analysed if it was characterised by an age or gender effect. The only study known to have undertaken such an analysis is Takao et al. (2011a), which did not find any significant effect. Hence, no age or gender effect in lateralization has been shown in the OR so far.

We found a significant hemisphere effect in all DTI metrics (FA, MD, λ_∥_ and λ_⊥_). We observed a left lateralization in FA and for the first time a significant gender effect with respect to this lateralization, females demonstrating more left lateralization than males. Interestingly, this lateralization was not found to be dependent on age. We also observed left lateralization of λ_∥_. This finding and the fact that λ_⊥_ was shown to be higher in the left OR (in addition with decreasing λ_⊥_ with age in the right OR) suggest lower axon density in the left hemisphere. For example, the findings of Schwartz et al. (2004) showed that anisotropy and parallel and perpendicular diffusivities were negatively correlated to axon counts and axon spacing in histology data of rat spinal cord.

#### Sexual dimorphism

With respect to sexual dimorphism in specific WM tracts, positive findings are reported in the literature notably for the cingulum (Schneiderman et al. 2007; Bonekamp et al. 2007; Lebel and Beaulieu 2011), inferior longitudinal fasciculus (Eluvathingal et al. 2007; Bava et al. 2011; Choi et al. 2010a), superior longitudinal fasciculus (Bava et al. 2011; Lebel and Beaulieu 2011), right arcuate fasciculus (Schmithorst et al. 2008), splenium of the corpus callosum (Schmithorst et al. 2008; Lebel and Beaulieu 2011), fornix (Perrin et al. 2009) and corticospinal tract (Perrin et al. 2009; Schmithorst et al. 2008; Bava et al. 2011; Lebel and Beaulieu 2011), while discrepancies exist for other tracts. To our knowledge, there is no report of sexual dimorphism for DTI metrics within the OR.

In addition to the gender effect already discussed in the lateralization of FA, we also found for the first time sexual dimorphism for λ_∥_ in the right hemisphere with this metric being lower for females than males. Also in the same hemisphere, MD was shown to significantly decrease with age for males, but no such effect was found for females. This is similar to what has been found recently in other WM tracts by Clayden et al. (2011). It has been shown that WM volume increases more rapidly in males than females (Giedd et al. 1999; De Bellis et al. 2001; Lenroot and Giedd 2006), especially in the occipital lobe and may be more related to increasing axonal diameters than myelination (Perrin et al. 2008). The results we obtained would suggest that the same process is occurring within the OR, as shown by decreasing MD whilst lower values of λ_∥_ in females also supports the conclusions of Perrin et al. (2009) that WM may be more dense and with axons of smaller diameter in females.

### Neuroanatomical mapping

The most common type of epilepsy surgery is temporal lobectomy (Berg 2011). A notable risk during this operation is damaging the anterior portion of the OR, which can result in upper quadrantanopia (visual field defect in the contralateral upper visual field). However, the exact extent of this part of the OR, namely ML, has not previously been documented in children and adolescents in vivo. It is difficult to determine this extent due to the numerous neighbouring tracts present in this area, making it difficult to precisely dissect the OR ex vivo without severing it (Yasargil et al. 2004). Due to the variation in extent of ML between individuals and the frequency of temporal lobectomy surgery, in vivo mapping of the OR would prove particularly useful for neurosurgical planning. Tractography offers such an opportunity but suffers from the difficulty of validation due to the lack of within subject “ground truth”. Several tractography studies aimed at validating their results either qualitatively (see for example Catani et al. 2003; Fernandez-Miranda et al. 2008; Staempfli et al. 2007) or quantitatively by comparing the mean and range (or standard deviation) of their OR measurements with the ones obtained with dissection studies previously quoted, as is the case for Chen et al. (2009), Nilsson et al. (2007), Sherbondy et al. (2008), Yamamoto et al. (2005) and Yogarajah et al. (2009).

Studies based on conventional MRI showed that there was no significant change in the total cerebral volume after the age of 5 years (Giedd et al. 1996; Reiss et al. 1996; Lebel et al. 2008), although the volume of internal structures varies with time (Giedd 2004). The fact that no age effect was found for either the OR volume or the ML–TP distance appears to be consistent with the latter findings. We then hypothesise that the total number of fibers within the OR and its total size do not change significantly after the age of 5 years and thus allow for direct comparison with anatomical data from dissection studies in adults. This is desirable because of the lack of such dissection data of the OR in children and adolescents in the literature. Several studies found marked individual variations, notably Burgel et al. (1999, 2006), Choi et al. (2006), Ebeling and Reulen (1988), Peuskens et al. (2004), Pujari et al. (2008) and Rubino et al. (2005). The only known report of gender differences in the literature for dimensions of the OR was by Toosy et al. (2004) in an in vivo tractography study of 22 subjects (7 women and 15 men with mean age 29.6 ± 6.3 years) which described a gender effect for volume, being greater for females. This result was obtained after normalisation to a common template and there was no discussion of sexual dimorphism in absolute volume. No interhemispheric asymmetry was found except by Burgel who reported (after non-linear registration to a common reference brain) a left lateralization of the OR volume in his early work (Burgel et al. 1999), though not endorsed in his later work on the same subjects (Burgel et al. 2006) in which the reported asymmetry is limited to higher variability of the OR in the left hemisphere.

With respect to our reconstruction of the OR, the fact that the seed, waypoint and exclusion ROIs, and the tractography, were defined in native subject space had the advantage of providing the OR reconstruction intrinsic to each subject and avoiding the potential spatial errors typically introduced by the use of tracts or ROIs extracted from an atlas or group template after registration and its associated spatial transformations. The evaluation of accuracy is a major challenge of tractography and, to the best of the visual assessment of the authors, the reconstructed tracts complemented well both the surrounding anatomy and the displayed DTI information (notably each voxel main diffusion direction). The high inter-subject variability found in the extent of ML in the present study is consistent to what was found in dissection studies (Ebeling and Reulen 1988; Peuskens et al. 2004; Rubino et al. 2005; Choi et al. 2006; Pujari et al. 2008) and our results (cf. Table 1) are also similar to in vivo tractography studies in adult series such as Yogarajah et al. (2009) which reported ML–TP ± STD [min, max] as 34 ± 1 [24, 41] mm in the left OR and as 36 ± 1 [32, 47] mm in the right OR. However, the measured ML–TP distances were systematically greater than reported in dissection studies. A possible explanation could be that the tractography algorithm failed to reconstruct the most anterior part of ML, which is plausible due to the limited angular resolution provided by the 20-gradient direction DTI sequence, or that known structural changes occurring in the brain during and post-fixing in dissection studies were at the origin of the discrepancies. Any tractography approach employed is subject to the limited spatial resolution of the DTI acquisition which in this case provided 2.5 mm isotopic voxels. This can clearly be improved upon at higher field strengths but at the expense of more severe susceptibility distortions which need to be circumvented at the acquisition level or corrected for in post-processing.

**Fig. 13:**
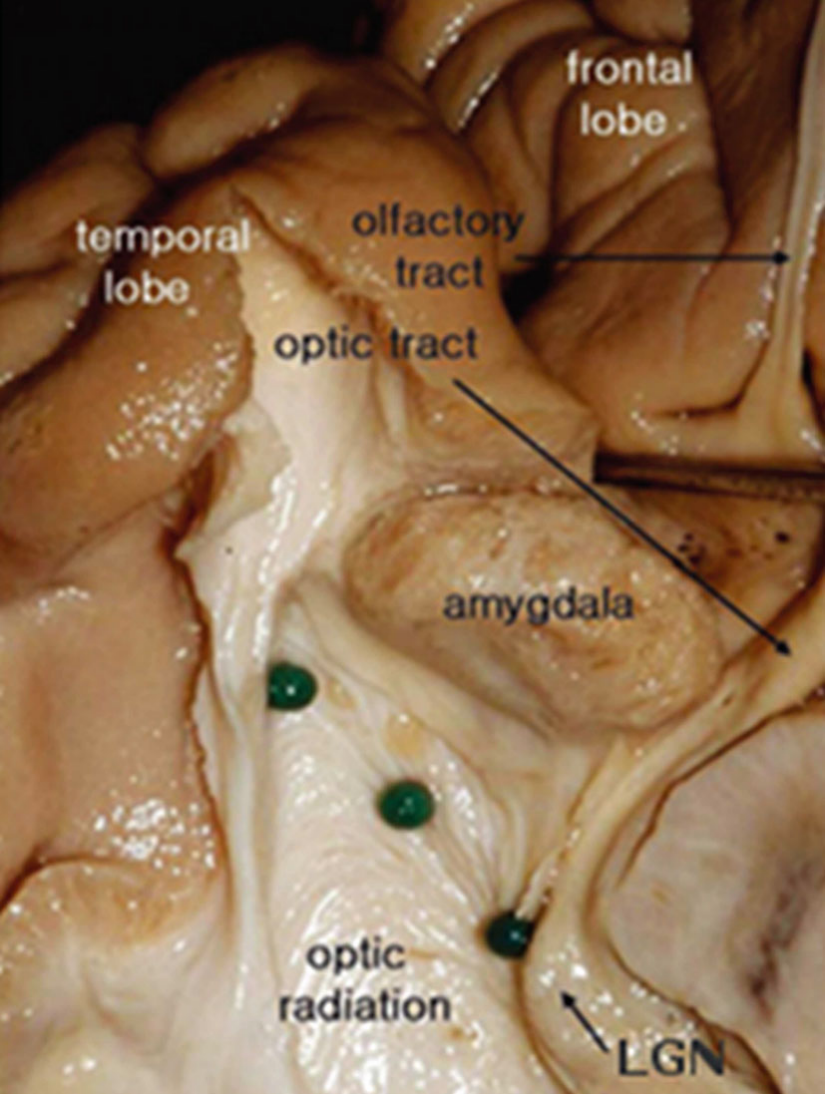
Position of the amygdala with respect to the OR. The location of the amygdala might explain the sexual dimorphism observed in the ML–TP distance. Reproduced with permission from Choi et al. (2010b)

We observed for the first time to our knowledge differences in ML–TP distances in the left hemisphere between males and females. This observation together with the documented mean values of ML–TP and their variability reflected in the standard deviation may be useful considerations for the neurosurgical planning of temporal lobectomy in children and adolescents. A possible explanation for this gender effect could be that it is related to the size of the amygdala, known to be larger in males (Giedd et al. 1996; Goldstein et al. 2001), which could play a role in restraining the extent of the OR in this gender as shown in Fig. 13.

The absolute values of the volume of the OR were similar to previous reports using tractography in adults (Toosy et al. 2004; Hernowo et al. 2011). We report for the first time significant right lateralization in OR volume (*p* < 0.00000005). As the OR is “sandwiched” between the lateral wall of the lateral ventricle on one side and other white matter structures on the other, the known greater volume of lateral ventricles in the left hemisphere (Giedd et al. 1996) might be a factor relating to the observed right lateralization.

## Conclusion

This study aimed at investigating both the structural maturational and anatomical dimensions of the OR in normally developing children and adolescents.

The OR is a white matter tract known to be myelin mature by the age of 3 years and the DTI metrics measured within the reconstruction obtained with tractography—namely FA, MD and λ_⊥_—demonstrated that maturational changes of the OR microstructure are still ongoing until at least adolescence. DTI metric differences according to gender and hemisphere were also observed. Asymmetry was shown for all metrics, all higher in the left hemisphere. Sexual dimorphism was demonstrated in the right hemisphere with λ_∥_ lower in females and MD decreasing with age for males, while no significant effect was found for females. Furthermore, we demonstrated gender effects in the lateralization of FA, with females being more left-lateralised than males.

We documented for the first time measures of the anterior extent of the OR—the ML–TP distance—in a cohort of children and adolescents and showed that the known high variability of this extent in adults was also present in this younger age group. Both ML–TP and the OR volume were demonstrated to be asymmetric, the volume being right lateralised, whilst for males ML–TP was larger in the left hemisphere. Sexual dimorphism was also found for ML–TP in the left hemisphere where it was found to be smaller in females. These observations are relevant for the neurosurgical planning of children and adolescents with intractable epilepsy being considered for temporal lobectomy.
